# Immunological effects of convalescent plasma therapy for coronavirus: a scoping review

**DOI:** 10.1186/s12879-021-06981-0

**Published:** 2021-12-24

**Authors:** Behnaz Esmaeili, Shahnaz Esmaeili, Zahra Pourpak

**Affiliations:** 1 Immunology, Asthma and Allergy Research Institute (IAARI), Tehran University of Medical Sciences, Tehran, Iran; 2 Diabetes Research Center, Endocrinology and Metabolism Clinical Sciences Institute, Tehran University of Medical Sciences, Tehran, Iran

**Keywords:** Coronavirus, COVID-19, Convalescent plasma therapy, Plasma therapy

## Abstract

**Background:**

Preliminary studies revealed the safety and effectiveness of convalescent plasma (CP) therapy for patients with coronavirus. In this study, we aimed to evaluate and summarize the available evidence on CP therapy, identify the research gap regarding the immunological response to CP therapy and pave the road for future studies.

**Methods:**

This study was conducted according to the Hilary Arksey and Lisa O’Malley framework. To find out the relevant studies, we searched PubMed, Scopus and Embase databases up to 30th May 2021. Data have been extracted according to three categories: (1) patients’ characteristics, (2) clinical and immunological responses to CP therapy and (3) pre-infusion screening of the CP samples.

**Results:**

A total of 12,553 articles were identified. One hundred fifty-four studies met the inclusion criteria for full-text review. More than half of the included studies (112 studies, (75.6%)) concluded satisfactory outcomes and or safety of CP infusion in patients. Results of studies showed the efficacy of CP therapy in clinical improvement (101 studies), decreasing in the level of inflammatory factors (62 studies), elimination or decreasing in viral load (60 studies), and induction or increase in antibody response (37 studies). Despite these promising results, the results of the 49 studies revealed that CP therapy was ineffective in the survival of patients, clinical improvement, viral infection elimination or decrease in the inflammatory factor levels. Furthermore, the adaptive immune response was evaluated in 3 studies. Information related to the pre-infusion screening for human leukocyte antigen/human neutrophil antigen (HLA/HNA) antibodies was not reported in most of the studies. Our gap analysis revealed that the influence of the CP infusion on the adaptive immune and inflammatory responses in patients with coronavirus needs further investigation.

**Conclusions:**

Based on the results of most included studies, CP infusion was safe and resulted in clinical improvement of patients and decreasing the viral load. The effect of the CP infusion on adaptive immune response and inflammatory cytokines in patients with coronavirus needs further investigation.

## Background

The novel member of human coronavirus (HCoV), severe acute respiratory syndrome coronavirus 2 (SARS-CoV-2) appeared in Wuhan, China and has spread quickly and resulted in a global pandemic. The disease caused by this virus was called Coronavirus disease 19 (COVID-19). By 18 June 2021, there have been 176,945,596 confirmed cases and nearly 3,836,828 deaths due to COVID-19 [1, 2]. The previous coronavirus outbreaks were severe acute respiratory syndrome (SARS) and Middle East respiratory syndrome (MERS); happening in 2002 and 2012, respectively [3, 4]. At the moment, various vaccines and some therapies (such as anti-viral treatments) are used to prevent and treat this disease, respectively [5, 6]. Although, the effectiveness of these strategies needs to be investigated further.

Convalescent plasma (CP) is the plasma obtained from recovered patients with an infectious disease and may contain antibodies against the pathogen, such as a virus, and acts as a therapeutic agent [7]. While it has received the United States Food and drug Administration (USFDA) approval to treat critically ill patients [8], uncertain and inconsistent results obtained from various clinical trials and case studies, have made it difficult to decide on the effectiveness of this therapy. Some of the studies showed that plasma therapy was not associated with a reduction in mortality or an improvement in the patients’ outcomes [9, 10]. However, other studies demonstrated the effectiveness of this treatment on patients’ recovery and prevention of the COVID19 progression [11, 12].

In addition to primary studies, the effectiveness of CP therapy in patients with coronavirus has been investigated by numerous systematic reviews and meta-analyses. While some of these studies showed that this treatment was ineffective in the treatment of patients with COVID-19, other investigations considered this method to be effective [13–16]. Moreover, several knowledge gaps, such as patient’s eligibility, dose and frequency of CP infusion have been identified in the clinical application of the CP in patients with COVID-19 that need to be addressed [17].

Considering the controversial results of these studies, further detailed investigations are required. Scoping review is a type of secondary study that can be used to identify the extent and range of studies in a particular field. It helps to find out the relevant research gaps and prioritize future research goals [18, 19].

To our knowledge, this study is the first scoping review that evaluates the clinical and immunological effects of CP therapy for coronavirus infections. Thus, we aimed to summarize studies related to CP therapy in patients with coronavirus, find out associated knowledge gaps, and prioritize future studies focusing on clinical and immunological responses to CP therapy. We also summarized the data of pre-infusion screening of human leukocyte antigen/human neutrophil antigens (HLA/HNA) antibodies in the CP samples.

## Methods

This scoping review was conducted based on the framework offered by the Hilary Arksey and Lisa O’Malley framework [18] which is composed of the following five stages: (a) identifying the research questions, (b) identifying relevant research studies, (c) studies selection, (d) extraction and charting the data, and (e) summarizing, analyzing and reporting the results.

### Identifying the research question

We tried to answer the following questions:

- What are the extent and range of the relevant studies?
- What are knowledge gaps related to the topic?
- Which items have been investigated regarding the evaluation of the clinical and immunological responses to CP therapy?
- What are the necessitates required to improve future relevant research?

### Literature search strategies

To obtain and identify the relevant publications, we searched three databases including PubMed, Scopus and Embase up to 30th May 2021. Following keywords and phrases have been used in this search: “convalescent plasma therapy, plasma therapy, serum therapy, hyperimmune immunoglobulin, passive immunization, passive antibody transfer, coronavirus immune plasma, human anti-SARS-CoV-2 plasma, coronavirus, Middle East respiratory syndrome coronavirus (MERS), severe acute respiratory syndrome (SARS), 2019 novel coronavirus disease, COVID-19, SARS-CoV-2 infection, MERS coronavirus, SARS-CoV, and MERS-CoV”.

### Identification and selection of relevant studies

**Fig. 1:**
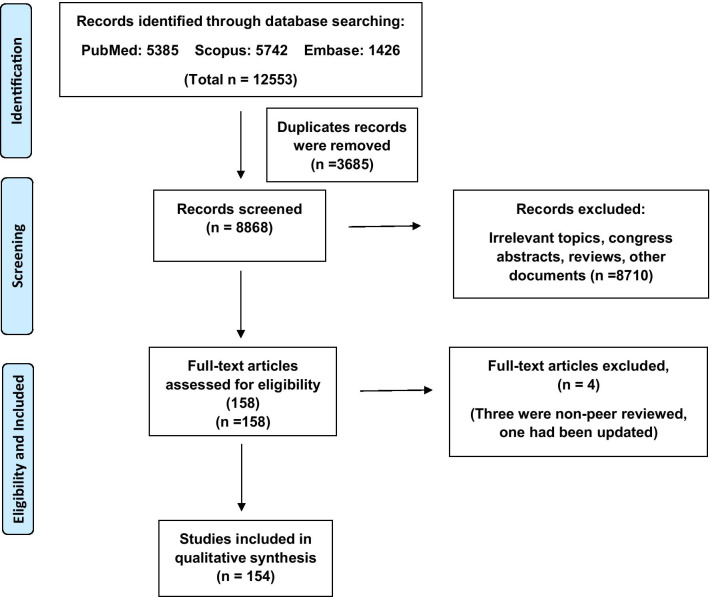
Flow chart diagram of the database search and final full texts included in this scoping review

To find out duplications, all the retrieved articles were imported to EndNote. Two reviewers (B.E and Sh.E) independently screened the titles and abstracts of articles based on the following eligibility criteria and any disagreement was resolved through discussion and consensus. In addition, full-text articles for the included publications were reviewed against the criteria by reviewers. According to the document type, we included original articles on human subjects regardless of the study design and participants. All types of reviews, congress abstracts, non-peer-reviewed, and irrelevant studies were excluded. Among grey literature, only letters reporting patients’ outcomes were included. Only English language publications were included. We also reviewed the reference list of the included articles. We presented the search results by a PRISMA (Preferred Reporting Items for Systematic Reviews and Meta-Analyses) flow diagram (Fig. 1).

### Data extraction from the included studies

Extracted data included date of publication, country of study, level of evidence, sample size, patients’ characteristics (sex, age, laboratory findings, clinical symptoms, level of anti-coronavirus antibodies and the immune response before and after CP therapy), type of coronavirus, adverse events, response to plasma therapy and HLA/HNA antibody screening of CP samples.

### Collating, summarizing and reporting the results

Descriptive statistics were used to assess the obtained results. Data were summarized and reported in the following categories: patients’ characteristics, response to CP therapy and the pre-infusion screening of the HLA/HNA antibodies in the CP samples and the adverse events.

## Results

A total of 12,553 articles were included in this scoping review. After removing the duplicates, a total of 8868 records were screened for titles and abstracts. Of these, 8710 records were excluded for these reasons: irrelevant topics, reviews, congress abstracts or unavailability and other documents. A total of 158 records were selected for full-text evaluation. Of theses, four articles were excluded from the final evaluation (3 were non-peer reviewed papers and one article had been updated). Finally, 154 articles were included for full-text evaluation. Screening processes and articles identification are summarized in Fig. 1.

The general characteristics of the included studies are summarized in Table 1. Among the selected articles, 27 were clinical trials and 112 were case reports, case series, or retrospective/observational studies. The data from 15 letters and communications were also included in this review. Most studies were conducted in the USA (n = 41), China (n = 24), India (n = 11), Turkey (n = 9), and Iran (n = 6). Most of the reports (n = 150) were from patients with COVID-19 (97.4% of the included studies). The remaining four studies were conducted on patients with SARS and MERS.

**Table 1:** Summary of characteristics of included studies, total (154)

	Number of studies	Continent**/**Countries (number of studies)
Publication year		
Before 2019	4	North America/USA (41), Canada (1)
2019–2020	81	South America/Mexico (2), Argentina (3), Colombia (1),
2020–2021	69	Cuba (1), Chile (1), Brazil (3)
Publication type		Europe/France (4), Italy (5), Poland (6), Greece (1), UK (2)
Case/case series	65	Austria (1), Germany (3), Netherlands (1), Spain (2), Russia (1)
Other observational studies	47	Romania (1), Croatia (1), Belgium (1)
Clinical trials	27	Asia/China (24), India (11), Turkey (9), Iran (6), Korea (4)
Letter/Communications	15	Hong Kong (1), Taiwan (1), Iraq (2), Qatar (1), Kuwait (1),
Type of coronavirus studied		Bahrain (1), Nepal (1), Oman (2), Israel (3), United Arab
SARS-Cov2	150	Emirates (2), Saudi Arabia (1), Lahore (1)
SARS	2	Africa/Egypt (1)
MERS	2	

### Clinical symptoms and laboratory findings of patients

Patients included both females and males of different ages; ranging from 9-months to 100 years. The sample size ranged from one to 20,000 participants. In most of the included studies, infected patients were identified by a positive viral molecular test as a routine confirmation test. Serologic testing and identifying the presence of antibodies against SARS, MERS, and COVID-19 has been assessed in 46 studies (29.8%). Of these, antibody levels were low or undetectable in 17 studies. In one study, data related to the patient’s antibodies were used for patient’s selection in which patients with high titer of S protein-RBD-specific (receptor binding domain) IgG antibody (≥ 1:640) were excluded. In 112 out of 154 included studies (72.7%), CP therapy has been conducted in severe or critically ill patients. In 24 (15.5%) and 11 (7.1%) studies CP therapy was performed in moderate to severe and mild patients. In 41 studies (26.6%), CP therapy has been used in patients after failure of other treatments and with clinical deterioration of patients. Generally, in most studies (97 out of 154) (62.9%) infusion of CP was performed as a combination therapy. The time of patient’s admission varied from the disease onset to 88 days after symptoms onset. Depending on the clinical status of patients and antibody response, one, two or multiple infusions have been performed.

The most commonly reported clinical symptoms of patients were fever, low levels of oxygen, cough, dyspnea and lung infiltration or abnormality. The most laboratory abnormalities found in patients were lymphopenia, increased levels of C-reactive protein/erythrocyte sedimentation rate (CRP/ESR), increased lactate dehydrogenase (LDH) level, high IL-6 level, high ferritin and increased d-dimer level. A summary of clinical symptoms and laboratory findings reported in the included studies are presented in Table 2.

**Table 2:** Summary of clinical symptoms and laboratory findings of patients with COVID19 reported in the included studies are listed, (total: 154)

Clinical symptoms	Number of studies (%)	Laboratory findings	Number of studies (%)
Fever	90 (58.4%)	Lymphopenia	72 (46.7%)
Low levels of oxygen O_2_	94 (61%)	Increased CRP/ESR	98 (63.6%)
Cough	74 (48%)	Increased LDH	41 (26.6%)
Pneumonia	49 (31.8%)	Increased IL-6	42 (27.2%)
Dyspnea	76 (49.3%)	Increased ferritin	49 (31.8%)
ARDS	24 (15.5%)	Increased d-dimer	46 (29.8%)
Lung abnormality	70 (45.4%)		
Chills	7 (4.5%)		
Diarrhea	26 (16.8)		
Poor appetite	6 (3.8%)		
Fatigue	24 (15.5%)		
Sore throat	11(7.1%)		
Vomiting and nausea	18 (11.6%)		
Chest pain	8 (5.1%)		
Headache	16 (10.3%)		
Myalgia	24 (15.5%)		
MODS	8 (5.1%)		
Anosmia/ageusia	5 (3.2%)		
General weakness	6 (3.8%)		
Abdomen pain	4 (2.5%)		

*ARDS* acute respiratory distress syndrome, *MODS* multiple organ dysfunction syndrome

### Clinical and immunological response to CP therapy and main findings

More than half of the included studies (112 studies, (75.6%)) concluded satisfactory outcomes and or safety of CP infusion. The main findings showed the effectiveness of CP therapy in the survival rate, clinical and radiological improvement, pulmonary recovery, decrease in inflammatory markers, decrease in the mortality rate, decreases in the length of stay (LOS) in the intensive care unit (ICU), removal of viral infection, increase in antibody levels, and preventing the progression to the severe form. However, 24 studies (16.2%) concluded that CP treatment was not safe and failed to improve clinical symptoms, decrease inflammatory markers, or increase the survival rate.

**Fig. 2:**
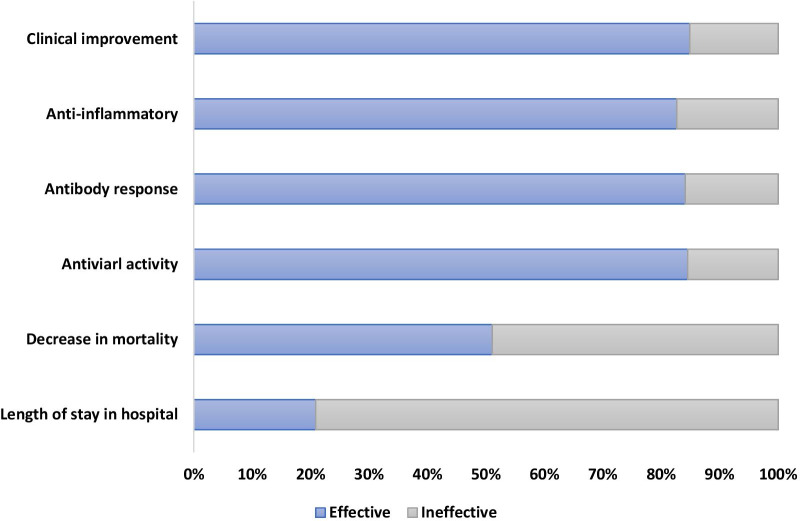
Bar graph showing the percentage of included studies depicting the efficacy or inefficacy of the CP therapy based on different categories

The response to the plasma therapy has been assessed through various items. Effect on the mortality rate, improvement in patients’ clinical symptoms, reducing the inflammatory factors, elimination or reducing the viral load, and induction or increasing the antibody response in the patients and safety are some of the investigated items, Fig. 2.

Results of 101 studies (68.2%) showed the efficacy of CP infusion in clinical improvement. However, results of 18 (12.1%) studies showed no significant effect of CP therapy in clinical improvement. A decrease in inflammatory factors or an increase in lymphocyte count has been reported in 62 studies (41.8%). However, the results of 13 studies (8.7%) showed no significant change in the CRP and interleukin-6 (IL-6) levels or lymphocyte count. The efficacy of the CP therapy in decreasing the viral load and/or eliminating the viral infection was reported by 60 studies (40.5%). However, the results of 11 studies (7.4%) showed no significant or no change in the tests. Also, the results of the 37 studies (25%) revealed that treatment with CP was effective in increasing antibody levels. However, 7 studies (4.7%) showed no significant change in the antibody levels. The decrease in mortality was reported in 24 studies (16.2%); while 23 studies (15.5%) reported no significant change in mortality rate. A decrease in hospital (LOS) was reported in 5 studies (3.3%). However, 19 studies (12.8%) showed no significant difference or longer hospital LOS.

The number of included studies for evaluation of the response to CP therapy is summarized in Table 3.

**Table 3:** Summary of the number of included studies for evaluation of the response to CP therapy categorized into different groups

Responses to CP therapy	Sub categories		Number of studies/(%)
Clinical symptoms	Fever/cough	Improvement	29/5
		NC/NS	1/1
	Chest	Improvement	45
		NC/NS	2
	SOFA	Improvement	4
		NC/NS	3
	PaO_2_/FiO_2_, SaO_2_	Improvement	53
		NC/NS	6
	O_2_ withdraw, ARDS resolution, Ventilation support	Improvement	26
		NC/NS/need	8
Inflammatory response	IL-6 level	Decrease	16
		NC/NS Increase	11
	CRP level	Decrease	55
		NC/NS Increase	10
	Ferritin	Decrease	14
		NC/NS Increase	10
	D-dimer	Decrease	13
		NC/NS Increase	7
	LDH	Decrease	10
		NC/NS Increase	6
Immunologic response	Antibody response	Increase	37
		NC/NS	7
	Lymphocyte	Increase	28
		NC/NS	6
	T cell response	–	3
Antiviral response (Load of virus)	Decrease		60
	NC/NS Remained positive		11

*NC* not change, *NS* not significant, *ARDS* acute respiratory distress syndrome, *(PaO*_*2*_*)/(FiO*_*2*_*)* partial pressure of oxygen/oxygen concentration. *SaO*_*2*_ Arterial oxygen saturation

### Pre-infusion screening of the CP samples for HLA/HNA antibodies and the adverse events

Pre-infusion screening of the HLA and HNA antibodies and virus inactivation was reported in 14 studies (9%). Eighty-three studies (53.8%) showed no serious adverse reactions following CP infusion. Seven studies (4.5%) reported transfusion-related acute lung injury (TRALI). Non-severe febrile/non-hemolytic transfusion reactions and allergic reactions were observed in 28 studies (18.18%).

## Discussion

In this scoping review, data related to 154 articles on CP therapy in patients with coronaviruses have been summarized and the clinical and immunological responses to treatment have been evaluated in detail.

Controversial results were obtained from numerous systematic reviews conducted on the safety and efficacy of CP therapy in patients with COVID-19. While some systematic reviews showed that CP therapy was clinically effective and could be considered as concomitant therapy with other drugs used for the treatment of COVID-19 [14, 20], a Cochran review stated that there was still doubt regarding the benefit and effectiveness of CP therapy in patients [21].

Based on the results of this scoping review, most included studies (most were case reports and observational studies) showed that CP infusion was effective in the clinical improvement of patients with coronavirus [22–25]. However, results of most randomized controlled trials (RCTs) showed non-significant differences in the clinical outcomes and/or mortality rate [10, 26–28]. To obtain a definite conclusion, further investigations are needed.

Numerous factors can affect the efficiency of treatment. In CP therapy these factors may be relevant to the patients as well as the plasma samples. Inflammatory factors have been proposed to be involved in response to CP therapy. In a recent study, lymphocyte counts were higher and inflammatory factors levels (like CRP and LDH) were lower in responder patients compared to the non-responder patients [29]. It has been revealed that CP acts as an immunomodulation agent and CP infusion may active anti-inflammatory pathways [30]. The anti-inflammatory effect of the CP has been reported for the treatment of other viral infections like Influenza A (H1N1) [31]. This review showed that there were conflicting results on the effects of CP therapy on the inflammatory markers. Among inflammatory markers, inflammatory cytokines have played an important role in COVID-19 pathogenesis and outcome [32]. According to the results of this scoping review, IL-6 was evaluated more than other inflammatory cytokines pre- and post-CP infusion. Controversial results were observed regarding the IL-6 level after CP therapy that needs further investigation. Regarding the other cytokines, a limited number of studies revealed controversial results in tumor necrosis factor (TNF), IL-1, IL-2, IL-4, IL-5, IL-6, IL-8, IL-10, IL-12p70, IL-17 and interferon-gamma (IFNγ) levels after CP therapy. Of 6 studies, the results of two RCTs showed a decrease in TNF and IFN, IFN-γ-inducible protein-10 (IP-10) and macrophage colony-stimulating factor (M-CSF) following CP therapy [33, 34]. However, other studies showed a non-significant, increase or variable results in the level of inflammatory cytokines [26, 29, 35, 36]. The influence of CP therapy on inflammatory cytokines needs further evaluation.

The immunologic effects of the CP therapy have been evaluated via numerous items. A decrease in lymphocytes count is one of the laboratory markers for COVID-19 that is correlated with disease severity and clinical outcome [37]. In patients with severe COVID-19, a decrease in lymphocytes count was negatively correlated to the serum inflammatory cytokines levels (IL-6, TNF) [38]. Our findings reveal that in the majority of the included studies treatment with CP resulted in lymphopenia improvement. Other innate immune cells (such as monocytes and neutrophils) and/or adaptive immune cell subsets were less evaluated. In a study, dynamic changes in monocytes and neutrophils counts were evaluated, the results of which showed no significant changes in the percentage of the monocytes. However, the neutrophil counts showed a significant drop 3 days after plasma infusion [29].

Among the included studies in our review, limited studies reported the eosinophil count in the patients with COVID-19. A decrease in eosinophil count has been reported in patients with COVID-19 [39–41]. The number of eosinophils returns to the normal level in the patients following clinical improvement [42]. Increase in the eosinophil count was correlated with better prognosis of the patients with COVID-19 [39]. It has been revealed that eosinophilia in patients with COVID-19 associated with lower level of CRP, lower requirement for hospitalization and ICU admission [43]. No significant difference in eosinophils count was observed in COVID-19 patients with diabetes mellitus (DM) receiving CP therapy compared to the patients with conventional treatment [44]. In a case report of a patient with COVID-19, eosinophilia was observed that following CP therapy and at the time of discharge the eosinophilia persisted [25]. The influence of the CP therapy on the eosinophil count needs further investigation. Evaluation of the adaptive human T cells response to CP therapy has been investigated in 3 studies. Accordingly, an increase in the number of CD4+ T cells, CD8+ T cells and CD3+ T cells as well as IFN production was reported [45, 46]. A decrease in activated/effector, effector memory CD4+ T cells, activated effector CD8+ T cells and naïve B cells were reported [47]. Considering the important role of lymphocyte cells in anti-viral immunity, the influence of the CP infusion on the adaptive immune and lymphocyte cells subtypes of patients with coronavirus needs further investigation.

Our review reveals that plasma therapy has been performed with no consideration to the certain eligibility criteria of patients including their clinical status or laboratory tests. Pre-infusion evaluation of patients for the presence of the antibodies against SARS-CoV, MERS-CoV and SARS-CoV2 was evaluated in a limited number of studies. In one study, data related to the patient’s antibodies were used for patient’s selection in which patients with high titer of S protein-RBD-specific (receptor binding domain) IgG antibody (≥ 1:640) were excluded from that study [10]. In this regard, the results of a study on CP therapy in patients with SARS revealed that patients who were PCR positive and seronegative for coronavirus at the time of plasma infusion had better outcomes [48]. The impact of positive antibody tests on the recovery of the patients needs further evaluation.

From 154 included studies, reports of 83 studies showed no serious adverse reactions following CP infusion. The preliminary results of a study by Joyner et al. suggested the safety of CP therapy on 5000 hospitalized patients with COVID-19 [49]. The results of an update performed on 20,000 hospitalized patients with COVID-19 showed a low incidence of all severe adverse events (SAEs) following CP therapy [50]. To minimize the risk of TRALI, the World Health Organization (WHO) published a guideline on the use of CP in a pandemic and emphasized on standardization of donor selection and the usage of male plasma as the preferred source (and/or from female donors tested for HLAs and HNAs antibodies) [51]. Pre-infusion HLAs and HNAs antibody screening has not been reported in many studies. Considering the high risk of lung injury in patients with COVID-19 and given that granulocyte-reactive and HLA antibodies have been found in the never allo-exposed Dutch donor population (both in females and males) [52], the occurrence of TRALI could lead to irreversible and even fatal outcomes in patients with COVID-19. Therefore, the requirement of evaluating CP samples for the presence of HLAs and HNAs antibodies should be determined.

## Strengths and limitations of the study

This scoping review is a comprehensive analysis of the clinical and immunological responses to CP therapy in patients with coronavirus. Furthermore, a summary of pre-infusion screening of CP samples with detailed analysis has been performed that was not addressed in previous reviews. Additionally, the influencing factors on response to CP therapy were discussed.

The lack of high-quality studies led us to write this scoping review. The literature review was performed in only three databases (PubMed, Scopus and Embase) that may not be comprehensive enough. Not searching the gray literature is another limit of this paper. Additionally, quality assessment of the studies has not been done in this review.

## Conclusion

The main finding of the most included studies was the effectiveness of the CP infusion in clinical improvement of the patients and decreasing the viral load. Based on the results of most included studies, CP infusion was safe, with no severe adverse reactions. Regarding the inflammatory markers, CP therapy showed satisfactory outcomes in decreasing the level of some inflammatory markers like CRP. However, the influence of the CP therapy on IL-6 and other inflammatory cytokines needs further evaluation. The effect of the CP infusion on adaptive immune response in patients with coronavirus needs further investigation.

## Data Availability

The datasets used and/or analysed during the current study are available from the corresponding author on reasonable request.

## Abbreviations

ARDS: Acute Respiratory Distress Syndrome
CP: Convalescent plasma
COVID-19: Coronavirus disease 2019
HCoV: Human coronavirus
HLAs: Human leukocyte antigens
HNAs: Human neutrophil antigens
KTR: Kidney transplant recipient
LOS: Length of stay
MODS: Multiple organ dysfunction syndrome
MERS: Middle East respiratory syndrome
PaO_2_/FiO_2_: Partial pressure of oxygen/oxygen concentration
RBD: Receptor binding domain
RCT: Randomized control trial
SARS-CoV-2: Severe acute respiratory syndrome coronavirus 2
SARS: Severe acute respiratory syndrome
TRALI: Transfusion Related Lung Injury
